# Schizophrenia Patients Discharged on Clozapine Plus Long-Acting Injectable Antipsychotics From a Public Psychiatric Hospital in Taiwan, 2006–2021

**DOI:** 10.1093/ijnp/pyad053

**Published:** 2023-08-24

**Authors:** Ta-Chun Lin, Ching-Hua Lin

**Affiliations:** Department of Psychiatry, Kaohsiung Chang Gung Memorial Hospital, Kaohsiung, Taiwan; Kaohsiung Municipal Kai-Syuan Psychiatric Hospital, Kaohsiung, Taiwan; Department of Psychiatry, School of Medicine, College of Medicine, Kaohsiung Medical University, Kaohsiung, Taiwan; Department of Post-Baccalaureate Medicine, College of Medicine, National Sun Yat-sen University, Kaohsiung, Taiwan

**Keywords:** Schizophrenia, clozapine, long-acting injectable antipsychotics, rehospitalization, temporal trend

## Abstract

**Background:**

Some schizophrenia patients treated with clozapine experience an inadequate response and adherence problems. The purpose of this study was to compare time to rehospitalization within 6 months in schizophrenia patients discharged on 3 clozapine regimens. Additionally, the temporal trend of prescription rate in each group was also explored.

**Methods:**

Schizophrenia patients discharged from the study hospital from January 1, 2006, to December 31, 2021, (n = 3271) were included in the analysis. The type of clozapine prescribed at discharge was divided into 3 groups: clozapine plus long-acting injectable antipsychotics (clozapine + LAIs), clozapine plus other oral antipsychotics (clozapine + OAPs), and clozapine monotherapy. Survival analysis was used to compare time to rehospitalization within 6 months after discharge among the 3 groups. The temporal trend in the prescription rate of each group was analyzed using the Cochran-Armitage Trend test.

**Results:**

Patients discharged on clozapine + LAIs had a significantly longer time to rehospitalization than those on clozapine + OAPs or clozapine monotherapy. The prescription rates of clozapine + LAIs and clozapine + OAPs significantly increased over time, whereas the prescription rates of clozapine monotherapy significantly decreased.

**Conclusions:**

Compared with the clozapine + OAPs group, the clozapine + LAIs group had a lower risk of rehospitalization and a lower dose of clozapine prescribed. Therefore, if a second antipsychotic is required for patients who are taking clozapine alone, LAIs should be considered earlier.

**Significance Statement**

A portion of schizophrenia patients treated with clozapine have an inadequate response and adherence problems. Patients discharged on clozapine plus long-acting antipsychotics had a longer time to rehospitalization than those on clozapine plus other oral antipsychotics or clozapine monotherapy within 6 months after discharge. If adding a second antipsychotic is required for patients taking clozapine alone, LAIs should be considered earlier because of the better effectiveness than oral formulations.

## INTRODUCTION

Schizophrenia is a chronic, recurrent, and severe mental disorder. The mainstay of treatment for schizophrenia is antipsychotics. Long-term antipsychotic use is beneficial for improving symptoms and preventing relapse. Drug compliance is required to stabilize psychotic symptoms and prevent recurrence/relapse. However, approximately one-third of patients with schizophrenia do not respond adequately to antipsychotics (Elkis and Buckley, 2016). Treatment-resistant schizophrenia (TRS) is commonly defined as lack of response to 2 or more antipsychotic trials of adequate dose (i.e., at least 600 mg chlorpromazine equivalent per day) and duration (i.e., at least 6 weeks) (Howes et al., 2017).

Clozapine monotherapy has been regarded as the most effective treatment for TRS (Kane et al., 1988; Farooq and Taylor, 2011). Nonetheless, not all patients with TRS will respond to clozapine monotherapy. It is estimated that about 40% to 70% of patients with TRS do not respond to clozapine monotherapy with adequate dose and duration (Barber et al., 2017). The impacts on such patients include severe and persistent symptoms, relapses and hospitalizations, poorer quality of life, and higher health care costs, suggesting that additional treatment strategies are required to relieve enduring symptoms. Clinicians commonly try a combination with a second antipsychotic to enhance the clinical effects (Barber et al., 2017). Because clozapine has a weak dopamine D2 receptor blockade, combining clozapine with a second antipsychotic with strong D2 receptor antagonist properties may increase D2 receptor occupancy and therefore improve the clinical efficacy (Muscatello et al., 2014). Combining 2 antipsychotics may lead to higher plasma levels of the respective antipsychotic through pharmacokinetic interactions. Consequently, the antipsychotic effect will increase (Fleischhacker and Uchida, 2014). In addition, concomitant antipsychotics allow dose reduction in clozapine to reduce the adverse effects of clozapine (Nielsen et al., 2011).

Nonadherence is a major obstacle for effective treatment with clozapine, resulting in multiple relapses, rehospitalizations, and persistent psychotic symptoms (Morken et al., 2008). Potential adverse effects of clozapine, such as agranulocytosis, seizures, myocarditis, pneumonia, bowel obstruction, and metabolic syndromes, as well as the need for regular follow-up for monitoring of white blood cell counts may limit the use of clozapine (Remington et al., 2016; Farooq et al., 2019). It is estimated that rates of intentional nonadherence range from 23% to 55% during treatment (Taylor et al., 2009; Davis et al., 2014; Legge et al., 2016).

Long-acting injectable antipsychotics (LAIs) were originally developed with a long elimination half-life. They can maintain a relatively stable concentration in the bloodstream and were intended for patients with frequent relapses or a history of nonadherence to oral antipsychotic formulations. LAIs provide superior long-term outcomes by reducing the high rates of relapses/rehospitalizations (Correll et al., 2016; C. H. Lin et al., 2019). The prescription of LAIs has been recommended in all phases of treatment, especially in the setting of partial adherence or nonadherence (Malla et al., 2013). Therefore, clozapine plus LAIs (abbreviate as “clozapine + LAIs”) appears to be a promising option for patients with clozapine monotherapy failure or adherence problems (Mutlu et al., 2022).

Several studies have reported that, apart from reducing nonadherence, clozapine combined with LAIs can be more effective. Clozapine + LAIs can decrease the risk of relapse and rehospitalization, thereby having positive effects on the clinical course and outcomes. Furthermore, it appears that lower doses of clozapine are required for patients receiving clozapine + LAIs (Kim et al., 2010; Souaiby et al., 2017; Bioque et al., 2020; Grimminck et al., 2020; Caliskan et al., 2021; Chakrabarti, 2021; Joo et al., 2022). However, a nationwide cohort study from Finland (Tiihonen et al., 2019), including 62 250 schizophrenia patients with 20 years or less of follow-up time, revealed that clozapine + LAIs did not have a significant effect on reducing rehospitalization compared with clozapine monotherapy (adjusted hazard ratio [HR]  = 1.01; 95% CI = .85–1.21).

Once the patients begin outpatient treatment, they may encounter lifestyle changes, nonadherence, and differences in the level of psychosocial support and supervision that may contribute to risk of relapse/rehospitalization (Grimminck et al., 2020). Rehospitalization is clinically indicated for patients who present with a relapse/recurrence of significant psychotic symptoms, dangerous or violent behavior, or deteriorated functioning and do not adequately respond to outpatient treatment (Lehman et al., 2004). Rehospitalization is commonly used as a proxy for relapse (Olivares et al., 2013).

A recent multicenter, observational, naturalistic, retrospective, 6-month mirror-image study (Bioque et al., 2020) has found that after 6-month treatment with clozapine + LAIs, patients showed a significant decrease in the number of hospitalizations compared with the previous 6-month period using clozapine monotherapy. However, in such mirror image studies, each participant serves as their own control, and observed changes from pre- to post-association introduction may lead to regression toward the mean (Fagiolini et al., 2017; Kishimoto et al., 2021). To explore the effectiveness of clozapine + LAIs using a retrospective cohort design for comparison, the purpose of this study was to calculate time to rehospitalization within 6 months in schizophrenia patients discharged on clozapine + LAIs, clozapine plus other oral antipsychotics (abbreviated as clozapine + OAPs), or clozapine monotherapy. We also evaluated the temporal trend in the prescription rate of each group at discharge from a public mental hospital during a 16-year period (2006–2021).

## METHODS

### Ethics

The current study was approved by the Kai-Syuan Psychiatric Hospital Institutional Review Board and was carried out in accordance with both the Declaration of Helsinki (2013) and Taiwan’s national legislation (Human Subjects Research Act, Taiwan). Written informed consent was exempted due to the use of anonymous and deidentified electronic data.

### Participants

This was a retrospective cohort study conducted at Kaohsiung Municipal Kai-Syuan Psychiatric Hospital, which is a public mental hospital in Kaohsiung City in southern Taiwan. The hospital includes 821 psychiatric beds, and the city has a population of around 2.72 million people and 2092 psychiatric beds.

Patient data were extracted retrospectively by reviewing charts and electronic medical records. The participants were inpatients who met the DSM-IV-TR or DSM-5 criteria for either schizophrenia or schizoaffective disorder (APA, 2000, 2013) and were discharged on clozapine from January 1, 2006, to December 31, 2021. Diagnoses were made by board-certified psychiatrists and were supported by clinical observations, interviews by the staff, information provided by main caregivers, and past medical records.

The type of clozapine prescribed at discharge was classified into 3 groups: clozapine + LAIs, clozapine + OAPs, and clozapine monotherapy. LAIs included first-generation LAIs (FGA-LAIs) and second-generation LAIs (SGA-LAIs). Anticholinergics used to treat extrapyramidal side effects (EPS) included biperiden and trihexyphenidyl. Daily doses of anticholinergics at discharge were converted into biperiden equivalents (Stefan Leucht et al., 2016).

### Follow-Up Procedures

After discharge, the patients were followed up in the outpatient clinic at Kai-Syuan Psychiatric Hospital. Generally, medications found to be effective for acute phase treatment will be continued during the maintenance phase of treatment (APA, 2021). The prescription status of antipsychotics at the time of discharge was extracted for analysis because discharge can be considered an indicator of patient stabilization (Roh et al., 2014).

The frequency of outpatient visits was based on clinical conditions and ranged from weekly to biweekly, monthly, or trimonthly. Apart from routine education and counseling, no other specialized care or therapy was provided. If a patient requires inpatient treatment, as decided by treating psychiatrist in the emergency room or outpatient department, they are admitted alone to the acute ward using the shared decision-making process. If an unoccupied bed is not available, there is a wait list for being admitted. If a patient has severe psychiatric symptoms that are so debilitating as to interfere with rational decision-making, pose a danger to self or others, and refuse to accept full-day hospitalization, involuntary hospitalization will be recommended by 2 board-certified psychiatrists. According to Taiwan’s Mental Health Act, involuntary hospitalizations must be reviewed and approved by the committee. Rehospitalizations within 6 months after discharge were reported.

### Statistical Analyses

The clinical characteristics were described as numbers and percentages, while averages were reported as means ± SDs. Pearson chi-squared test and 1-way ANOVAs were performed to compare clinical characteristics among groups. A post hoc test using the Bonferroni correction was applied to detect significance between 2 groups. There were 3 groups in the present study. For the Bonferroni correction, the *P* value of .05 was divided by the number of comparison groups, yielding a *P* value of .0167 (.05/3). Therefore, *P* < .0167 was considered significant for post hoc test between 2 groups (Waqar et al., 2021).

Kaplan–Meier survival analysis was used to determine time to rehospitalization within 6 months after discharge. The significance among group differences was measured using the log-rank test. The multivariate Cox proportional hazards regression model, with treatment (clozapine + LAIs, clozapine + OAPs, or clozapine monotherapy) as a fixed factor, was performed to adjust for variables that may affect time to rehospitalization within 6 months after discharge. Variables used in the model included sex, co-occurring alcohol abuse/dependence, co-occurring anxiety disorders/depression disorders, co-occurring hypertension, co-occurring diabetes, co-occurring hyperlipidemia, age (years), age at onset (years), number of previous hospitalizations, length of hospital stay (days), clozapine daily dose (mg), and biperiden equivalent daily dose (mg). Clozapine monotherapy was used as the reference treatment in the comparison of risk of rehospitalization. Adjusted hazard ratio for rehospitalization was estimated with 95% confidence intervals. A hazard ratio >1 indicated a higher likelihood of psychiatric rehospitalization compared with the reference treatment (i.e., clozapine monotherapy).

Study duration was divided into 8 periods: the first period (2006–2007), the second period (2008–2009), the third period (2010–2011), the fourth period (2012–2013), the fifth period (2014–2015), the sixth period (2016–2017), the seventh period (2018–2019), and the eighth period (2020–2021). This method was previously used (Moreno et al., 2007; Greil et al., 2012; Rhee et al., 2020; Lin et al., 2023).

The Cochran-Armitage trend test was performed to evaluate the statistical significance of the temporal trend in prescription rates of clozapine + LAIs, clozapine + OAPs, and clozapine monotherapy from the first period to the eighth period. Additionally, the prescription rates of FGA-LAIs and SGA-LAIs among patients discharged on clozapine + LAIs were also examined. All tests were 2-tailed, and the statistical significance level was set as *P < *.05 (except post hoc test using Bonferroni correction). The data were analyzed using SPSS version 27.0 (IBM Corp., Armonk, NY, USA), SAS 9.4 software (SAS institute Inc, Cary, NC, USA), and MedCalc (MedCalc Software, Belgium).

## RESULTS

A total of 18 840 schizophrenia patients were discharged during the study period. Among the 18 840 patients, 3271 patients discharged on clozapine + LAIs (n = 305, 9.3%), clozapine + OAPs (n = 1091, 33.4%), or clozapine monotherapy (n = 1875, 57.3%) were eligible for analysis (Table 1). A flowchart of the participant selection process is shown in supplementary Figure 1. The mean age was 45.3 ± 11.0 years, and 57.7% (n = 1886) were male. Among the 305 patients treated with clozapine + LAIs, 234 (76.7%) received FGA-LAIs (flupenthixol decanoate = 30.7% [n = 72], haloperidol decanoate = 69.3% [n = 162]) and 71 (23.3%) received SGA-LAIs (aripiprazole LAI = 7.0% [n = 5], risperidone LAI = 57.7% [n = 41], 1-month formulation of paliperidone LAI = 35.3% [n = 25]).

**Table 1.** Comparison of Clinical Characteristics Among Schizophrenia Patients Discharged on Clozapine + LAIs, Clozapine + OAPs, and Clozapine Monotherapy

Characteristic	Clozapine + LAIs (n = 305)	Clozapine + OAPs (n = 1091)	Clozapine monotherapy (n = 1875)	*P*
Sex, male, n (%)	199 (65.2%)	652 (59.8%)	1035 (55.2%)	**.001** ^*a*^
Co-occurring alcohol abuse/dependences– yes, n (%)	56 (18.4%)	154 (14.1%)	218 (11.6%)	**.002** ^*a*^
Co-occurring anxiety disorders/depression disorders– yes, n (%)	48 (15.7%)	137 (12.6%)	281 (15.0%)	.139^*a*^
Co-occurring hypertension– yes, n (%)	52 (17.0%)	181 (16.6%)	297 (15.8%)	.793^*a*^
Co-occurring diabetes– yes, n (%)	79 (25.9%)	254 (23.3%)	431 (23.0%)	.535^*a*^
Co-occurring hyperlipidemia– yes, n (%)	33 (10.8%)	96 (8.8%)	143 (7.6%)	.135^*a*^
Age (y), mean (SD)	43.7 (10.0)	44.6 (11.2)	46.0 (11.1)	**<.001** ^*b*^
Age at onset (y), mean (SD)	24.1 (9.0)	24.0 (10.0)	25.7 (10.7)	**.001** ^*b*^
No. of previous hospitalizations, mean (SD)	6.1 (6.5)	5.6 (6.0)	4.7 (5.5)	**<.001** ^*b*^
Length of hospital stay (d), mean (SD)	214.1 (366.8)	257.4 (464.4)	337.3 (551.5)	**<.001** ^*b*^
Clozapine daily dose (mg), mean (SD)	184.4 (109.1)	230.4 (132.3)	236.1 (111.4)	**<.001** ^*b*^
Biperiden equivalent daily dose (mg), mean (SD)	2.2 (3.0)	2.0 (3.1)	1.0 (2.5)	**<.001** ^*b*^

Abbreviations: LAIs, long-acting injectable antipsychotics; OAPs, other oral antipsychotics.

^*a*^Pearson χ^2^ test.

^*b*^One-way ANOVA. Bolded values are statistically significant.

Significant differences among groups were observed for sex, co-occurring alcohol abuse/dependence, age, age at onset, number of previous hospitalizations, length of hospital stay, clozapine daily dose, and biperiden equivalent daily dose (Table 1). *P* values of post hoc tests using Bonferroni correction are shown in Table 2. Patients discharged on clozapine monotherapy were significantly more likely to be female gender, less likely to have a co-occurring alcohol abuse/dependence, be older, have a later onset of psychosis, have fewer number of previous hospitalizations, stay longer in the hospital, and receive a lower biperiden equivalent daily dose of anticholinergic prescription compared with those received clozapine + LAIs or clozapine + OAPs. Meanwhile, the clozapine + LAIs group took a significantly lower clozapine daily dose than the clozapine + OAPs group (post hoc test, *P* <.001) and clozapine monotherapy (post hoc test, *P* <.001).

**Table 2.** *P* Values of Post Hoc Test Using Bonferroni Correction^*a*^

Characteristic	Clozapine + LAIs vs Clozapine monotherapy	Clozapine + LAIs vs Clozapine + OAPs	Clozapine monotherapy vs Clozapine + OAPs
Sex-male/female	**.001**	.083	**.0156**
Age (y), mean (SD)	**<.001**	.182	**<.001**
Age at onset (y), mean (SD)	**.007**	.869	**<.001**
No. of previous hospitalizations, mean (SD)	**<.001**	.197	**<.001**
Length of hospital stay (days), mean (SD)	**<.001**	.087	**<.001**
Clozapine dose (mg/d), mean (SD)	**<.001**	**<.001**	.232
Biperiden equivalent dose (mg/d), mean (SD)	**<.001**	.350	**<.001**

Bolded values are statistically significant.

^*a*^Statistical significance was set at *P* < .0167.

### Rehospitalization

**Figure 1.**
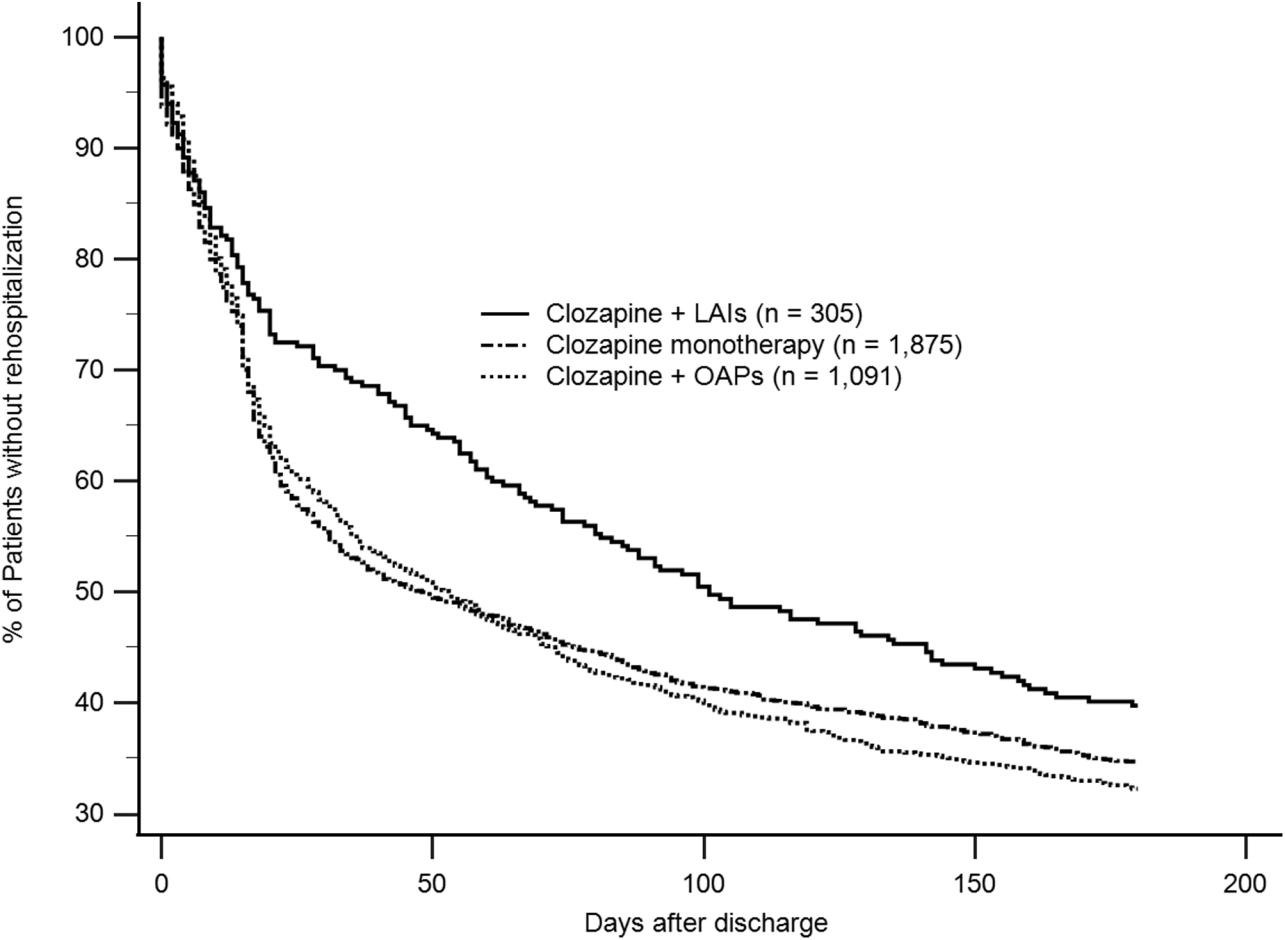
Time to rehospitalization for schizophrenia patients discharged on clozapine + LAIs (n = 305), clozapine + OAPs (n = 1,091), or clozapine monotherapy (n = 1,875) (log rank = 8.072 df = 2, *P* = .018).

The chi-square analysis indicated that patients discharged on clozapine + LAIs had a significantly lower rate of rehospitalizations (clozapine + LAIs, 55.4% [169/305]; clozapine + OAPs, 63.2% [689/1,091]; clozapine monotherapy, 60.9% [1,141/1,875]; χ^2^ = 6.138,df = 2, *P* = .046). The median time to rehospitalization differed significantly among the 3 groups: clozapine + LAIs, median time ±SE = 101.0 ± 17.1 days; clozapine + OAPs, median time ±SE = 53.0 ± 5.7 days; clozapine monotherapy, median time ±SE = 48.0 ± 5.3 days (log rank = 8.072, df = 2, *P* = .018) (Figure 1). After adjusting for other variables (i.e., sex, co-occurring alcohol abuse/dependence, co-occurring anxiety disorders/depression disorders, co-occurring hypertension, co-occurring diabetes, co-occurring hyperlipidemia, age, age at onset, number of previous hospitalizations, length of hospital stay, clozapine daily dose, and biperiden equivalent daily dose) using multivariate Cox proportional hazards regression analysis, the clozapine + LAIs group at discharge was still significantly associated with a longer time to rehospitalization (adjusted HR = 0.829; 95% CI = .703–.978, *P* = .026). The risk of psychiatric rehospitalization was 17.1% lower in the clozapine + LAIs group compared with the clozapine monotherapy group. In contrast, the risk of rehospitalization on clozapine + OAPs did not differ from that of clozapine monotherapy (adjusted HR = 1.032; 95% CI = .936–1.137, *P* = .527) (Table 3). Other variables associated with time to rehospitalization are shown in Table 3.

**Table 3.** Potential Factors Associated With Time (Days) to Rehospitalization in the Multivariate Cox Regression Analysis

Factors	B	Adjusted HR	95.0% CI	*P*
Treatment				
Clozapine + LAIs	−0.187	0.829	0.703–0.978	**.026**
Clozapine + OAPs	0.031	1.032	0.936–1.137	.527
Clozapine monotherapy—reference				.040
Sex (male vs female)	0.007	1.007	0.917–1.106	.879
Co-occurring alcohol abuse/dependences (yes vs no)	0.03	1.030	0.900–1.18	.666
Co-occurring anxiety/depression disorders (yes vs no)	0.095	1.100	0.966–1.253	.151
Co-occurring hypertension (yes vs no)	−0.013	0.987	0.852–1.145	0.867
Co-occurring diabetes (yes vs no)	0.128	1.137	1.005–1.285	**.041**
Co-occurring hyperlipidemia (yes vs no)	−0.098	0.907	0.771–1.067	.240
Age	0.016	1.016	1.012–1.021	**<.001**
Age at onset	−0.007	0.993	0.988–0.998	**.007**
No. of previous hospitalizations	0.034	1.035	1.028–1.042	**<.001**
Length of hospital stay (d)	0.001	1.001	1.0005–1.001	**<.001**
Clozapine daily dose (mg/d)	1.0002	1.0002	0.9998–1.001	.327
Biperiden equivalent dose (mg/d)	0.016	1.016	1.0002–1.032	**.048**

Abbreviations: CI, confidence interval; HR, hazard ratio; LAIs, long-acting injectable antipsychotics; OAPs, other oral antipsychotics.

Bolded values are statistically significant.

### Temporal Trend of Clozapine + LAIs Prescription

**Figure 2.**
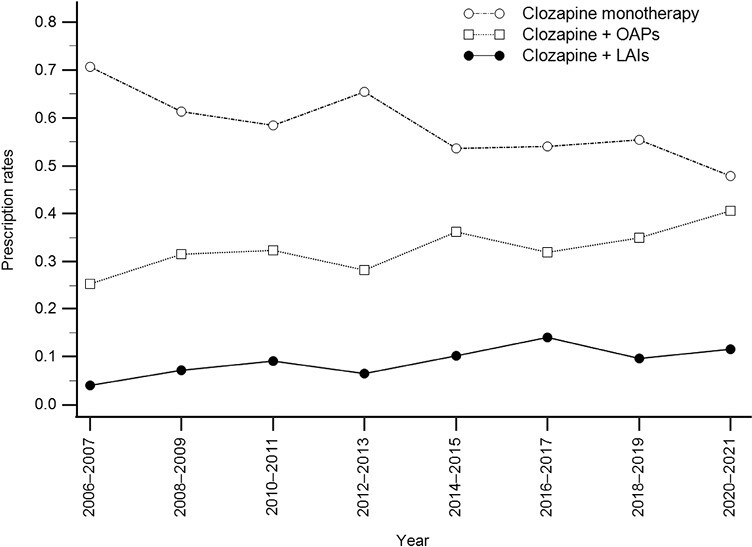
Temporal trends of the prescription rates in clozapine monotherapy, clozapine + OAPs, and clozapine + LAIs among schizophrenia patients at discharge.

The analysis of the Cochran-Armitage Trend test revealed a significant decreasing trend in the prescription rates of clozapine monotherapy (z = 6.582, *P < *.001) but a significant increasing trend in the prescription rates of clozapine + OAPs (z = 4.392, *P < *.001) and clozapine + LAIs (z = 4.075, *P < *.001) (Table 4). Figure 2 illustrates the prescribing trends for clozapine monotherapy, clozapine + OAPs, and clozapine + LAIs at discharge among all patients. Among patients discharged on clozapine + LAIs, the analysis of the Cochran-Armitage Trend test showed that the prescription rates of SGA-LAIs significantly increased over time. In contrast, FGA-LAIs use significantly declined from the first period to the last period (z = 5.746, *P < *.001) (Table 4).

**Table 4.** Temporal Trends in Rate of Patients Discharged on Clozapine + LAIs, Clozapine + OAPs, and Clozapine + Monotherapy, 2006–2021

Treatment	2006–2007	2008–2009	2010–2011	2012–2013	2014–2015	2016–2017	2018–2019	2020–2021	z	*P* ^*a*^
Patients discharged, n	276	346	364	439	403	415	471	557		
Clozapine monotherapy	195 (70.7%)	212 (61.3%)	213 (58.5%)	287 (65.4%)	216 (53.6%)	224 (54.0%)	261 (55.4%)	267 (47.9%)	6.582	**<.001**
Clozapine + OAPs	70 (25.4%)	109 (31.5%)	118 (32.4%)	124 (28.2%)	146 (36.2%)	133 (32.0%)	165 (35.0%)	226 (40.6%)	4.392	**<.001**
Clozapine + LAIs	11 (4.0%)	25 (7.2%)	33 (9.1%)	28 (6.4%)	41 (10.2%)	58 (14.0%)	45 (9.6%)	64 (11.5%)	4.075	**<.001**
Clozapine + FAG-LAIs	9 (81.8%)	25 (100.0%)	33 (100.0%)	23 (82.1%)	39 (95.1%)	36 (62.1%)	34 (75.6%)	35 (54.7%)	5.746	**<.001**
Clozapine + SGA -LAIs	2 (18.2%)	0 (0.0%)	0 (0.0%)	5 (17.9%)	2 (4.9%)	22 (37.9%)	11 (24.4%)	29 (45.3%)		

Abbreviations: FGA, first-generation antipsychotic; LAIs, long-acting injectable antipsychotics; OAPs, other oral antipsychotics; SGA, second-generation antipsychotic.

^*a*^Cochran-Armitage Trend test.

## DISCUSSION

The first finding of this study was that the clozapine + LAIs group was associated with a dose reduction of clozapine. This finding was consistent with other studies (Kim et al., 2010; Bioque et al., 2020). Therefore, the dose-related adverse effects of clozapine, such as tachycardia, hypotension, ileus, and seizure (Freeman and Oyewumi, 1997; Gurrera et al., 2022), could be decreased. Three mirror-image studies have evaluated the adverse effects of adding LAIs to clozapine in patients with TRS. One 6-month mirror-image study (Bioque et al., 2020) found that there was a significant decrease in the number and severity of adverse effects with clozapine + LAIs, leading to a decrease in the Udvalg for Kliniske Undersogelser total score. The authors concluded that the decrease in clozapine doses could be a potential mediator in the reduction of the number and severity of adverse effects. Another 1-year mirror-image study (Souaiby et al., 2017) reported that no major adverse effects occurred in terms of weight gain, agranulocytosis, hyperglycemia, and/or dyslipidemia after clozapine + LAIs use. The other 1-year mirror-image study (Caliskan et al., 2021) revealed no statistically significant change in the levels of neutrophil counts, total cholesterol, triglyceride, high-density lipoprotein, low-density lipoprotein, prolactin, and fasting blood glucose after adding LAIs to clozapine. The authors emphasized that the addition of LAIs to clozapine was generally well tolerated; however, continuous monitoring of possible adverse effects is still needed.

The second finding was that, according to the World Health Organization, the defined daily dose for clozapine is 300 mg/d (Stefan Leucht et al., 2016; WHO, 2023), which is higher than the average daily dose of clozapine in each group observed in the present study (Table 1). One possibility for this phenomenon is that Asians may have lower clozapine metabolism (Subramaniam et al., 2007; Faden, 2019). However, compared with patients discharged on clozapine monotherapy, those on clozapine + LAIs or clozapine + OAPs took a higher daily dose of biperiden equivalent (Tables 1 and 2). Anticholinergic agents are generally used to treat EPS. It is reasonable to expect that the combination strategies (i.e., combined with LAIs or OAPs) cause more EPS than clozapine monotherapy.

The third finding was that patients discharged on clozapine + LAIs had a longer time to rehospitalization than those on clozapine + OAPs or clozapine monotherapy within 6 months after discharge. This superiority remained statistically significant after adjusting for covariates (Table 3). An Asian nationwide, health insurance data–based study also found that clozapine + LAIs is associated with the lowest risk of psychiatric hospitalization compared with clozapine monotherapy (Joo et al., 2022). As mentioned above, nonadherence is common for patients treated with clozapine. Clozapine withdrawal will be associated with rebound psychosis, insomnia, agitation, aggression, and abnormal movements (Shiovitz et al., 1996; Ahmed et al., 1998; Stevenson et al., 2013). Concomitant use of LAIs can provide a pharmacologic “second-line of defense” against complete symptomatic exacerbation and withdrawal adverse events (Leung et al., 2014). Consequently, treatment outcomes are improved. In Table 3, relatively lower rates of co-occurring alcohol abuse/dependence (13.1%) were found among the participants. The rate of alcohol abuse/dependence among Taiwan Chinese is much lower than that among White people (Yang, 2002). Such a phenomena may be due to the marked sensitivity to alcohol and associated high levels of acetaldehyde, which acts as a biological protective factor (Hahn et al., 2006). In addition, a longer length of hospital stay was associated with a shorter time to rehospitalization (adjusted HR = 1.001; 95% CI = 1.0005–1.001, *P < *.001). Patients with a longer length of hospital stay reflect more chronicity, more severe symptomatology, a lack of effective treatment, or inadequate psychosocial support that may contribute to the increased risk of psychiatric rehospitalization (Lin et al., 2019a).

### Temporal Trend of Clozapine + LAIs Use

In this study, schizophrenia patients discharged on clozapine + LAIs and clozapine + OAPs significantly increased over time, while those on clozapine monotherapy significantly decreased from the first period to the last one. There are some possible reasons for such trends. First, as clozapine in some patients may take longer than the usual 4–8 weeks to reach clinical response (Fabrazzo et al., 2002; Nielsen et al., 2011), psychiatrists may add on a second antipsychotic (oral or LAI formulations) to speed up symptom reduction to meet the expectations of nursing staff, and patients and their families. Second, chronicity, treatment resistance, and comorbid conditions are more common among patients in public institutions (Patrick et al., 2006). In real-world clinical practice, especially in a setting such as public mental hospitals, psychiatrists will face schizophrenia patients with severe impulsive/violent behavior from time to time who have insufficient response to clozapine monotherapy. The prescriptions of clozapine + LAIs or clozapine + OAPs are associated with the management of more complex, more severe, and treatment-resistant cases (S. Leucht and Tiihonen, 2021). Third, the increasing prescription in clozapine + LAIs may perhaps reflect the psychiatrists gained experience and success with such regimen for patients with difficult-to-treat symptoms. In the clozapine + LAIs group, SGA-LAIs were not superior to FGA-LAIs in rehospitalization rate (SGA-LAIs = 54.9% [39/71]; FGA-LAIs = 55.6% [130/234]; χ^2^ = 0.009, df = 1, *P* = .926) and time to rehospitalization (SGA-LAIs, median time ± SE = 99.0 ± 44.1 days; FGA-LAIs, median time ± SE = 103.0 ± 17.3 days; log rank = 0.130, df = 1, *P* = .718) (data not shown in the table). The increase in SGA-LAIs use over time (Table 4) can probably be explained by their better tolerability. The biperiden equivalent daily dose was significantly greater in patient on FGA-LAIs (2.40 ± 3.15 mg/d) than those on SGA-LAIs (1.55 ± 2.24) (*P* = .012) (data not shown in the table). The results are hardly surprising, as SGA-LAIs cause fewer EPS than FGA-LAIs and may be more appealing to patients and clinicians as add-on agents to clozapine monotherapy (Miyamoto et al., 2002).

### Strengths and Limitations

There were several strengths in the current study. First, this was a long-term observational, retrospective study that allowed for a better assessment of real-world effectiveness. Second, hospitalization for schizophrenia is the largest amount of treatment costs and policy-relevant clinical outcome measure (Fitzgerald et al., 2007; Ascher-Svanum et al., 2010). Third, many potential factors were adjusted for, thus decreasing confounding. Fourth, clozapine was further grouped into the clozapine +LAIs, clozapine + OAPs, and the clozapine monotherapy to investigate the effectiveness and temporal trends among groups. Fifth, the large sample size (n = 3271) can provide statistical power to detect the primary association of interest.

Several limitations of this study should also be addressed. First, the observational study design is limited by selection bias. Second, the diagnoses of schizophrenia were based on clinical judgments and not standardized interviews reflecting DSM-IV-TR or DSM-5 criteria. Third, this cohort study was composed of patients who had just been discharged from a public psychiatric hospital, and the results may not generalize to patients from a general hospital or community. Fourth, many potential variables were not examined, such as hospitalization policy, symptom severity, functional impairment, medication adherence, and reasons for medication changes. Furthermore, in our study, it is not known whether clozapine was tried before LAI use. Fifth, not all patients included for analysis were insufficient responders to clozapine monotherapy. Clozapine can take 6 to 12 months to achieve full efficacy (Meltzer, 1992; Grimminck et al., 2020). Sixth, as aforementioned, clozapine + LAIs or clozapine + OAPs are commonly used for patients who do not respond adequately to clozapine monotherapy. Treating psychiatrists prescribe such combinations based on clinical necessity and their clinical experiences. Being a naturalistic study, the actual reason to prescribe clozapine + LAIs or clozapine + OAPs cannot be known. This reason may influence the rehospitalization rate. Seventh, clozapine plasma levels and detailed adverse effects among 3 groups were not available in this retrospective study. Finally, the use of an all-Taiwanese sample may limit generalizability of the findings to other races/ethnicities or countries.

## CONCLUSIONS

Schizophrenia patients discharge on clozapine + LAIs and those on clozapine + OAPs had comparable clinical characteristics with respect to sex, age, age at onset, number of previous hospitalizations, length of hospital stay, and biperiden equivalent daily dose. However, patients discharged on clozapine + OAPs were more likely to receive higher dose of clozapine and had a shorter time to rehospitalization than those on clozapine + LAIs. In contrast, clozapine + OAPs group did not decrease the risk of rehospitalization as compared with clozapine monotherapy (Tables 2 and 3). If adding a second antipsychotic is required for patients taking clozapine alone, LAIs should be considered earlier because of the better effectiveness than oral formulations. Long-term, controlled, and prospective follow-up studies are needed to explore clinical outcomes and tolerability using validity scales. These studies should be conducted to obtain more evidence-based support for the potential beneficial effects of clozapine + LAIs in the future. Further study should also consider using the time frame until the patients who did not require rehospitalization during the study period to compare the mean duration among the 3 groups.

## Supplementary Material

pyad053_suppl_Supplementary_Figure_S1

## Data Availability

The data will be shared on reasonable request to the corresponding author.
