# Supplementary material for: Schizophrenia Patients Discharged on Clozapine Plus Long-Acting Injectable Antipsychotics From a Public Psychiatric Hospital in Taiwan, 2006–2021
Source: Int J Neuropsychopharmacol. 2023 Aug 24;26(11):808–16. doi: 10.1093/ijnp/pyad053 (PMC10674076; doi:10.1093/ijnp/pyad053)
Supplement: pyad053_suppl_Supplementary_Figure_S1 [file pyad053_suppl_supplementary_figure_s1.docx]

18,840 schizophrenia patients discharged from 2006 to 2021

15,569 excluded

141 clinical trial

518 no antipsychotic use at discharge

14,910 receiving other antipsychotics

3,271 discharged on clozapine entered the analysis

clozapine + LAIs

n = 305

clozapine monotherapy

n = 1,875

clozapine + OAPs

n = 1,091

Supplementary Figure 1. Selection of subjects for the analysis.
